# Promoting Functional Health in Midlife and Old Age: Long-Term Protective Effects of Control Beliefs, Social Support, and Physical Exercise

**DOI:** 10.1371/journal.pone.0013297

**Published:** 2010-10-11

**Authors:** Margie E. Lachman, Stefan Agrigoroaei

**Affiliations:** Psychology Department, Brandeis University, Waltham, Massachusetts, United States of America; James Cook University, Australia

## Abstract

**Background:**

Previous studies have examined physical risk factors in relation to functional health, but less work has focused on the protective role of psychological and social factors. We examined the individual and joint protective contribution of control beliefs, social support and physical exercise to changes in functional health, beyond the influence of health status and physical risk factors in middle-aged and older adults. Given that functional health typically declines throughout adulthood, it is important to identify modifiable factors that can be implemented to maintain functioning, improve quality of life, and reduce disability.

**Methodology/Principal Findings:**

We conducted a national longitudinal study, Midlife in the United States (MIDUS), with assessments in 1995–1996 and 2004–2006, and 3,626 community-residing adults, aged 32 to 84, were included in the analyses. Functional health (Physical Functioning subscale of the SF-36) and protective factors were measured at both occasions. While controlling for socio-demographic, health status, and physical risk factors (large waist circumference, smoking, and alcohol or drug problems), a composite of the three protective variables (control beliefs, social support, and physical exercise) at Time 1 was significantly related to functional health change. The more of these factors at Time 1, the better the health maintenance over 10 years. Among middle-aged and older adults, declines in health were significantly reduced with an increased number of protective factors.

**Conclusion/Significance:**

Age-related declines in health were reduced among those with more protective factors up to a decade earlier in life. Modifiable psychological, social, and physical protective factors, individually and in the aggregate, are associated with maintenance of functional health, beyond the damaging effects of physical risk factors. The results are encouraging for the prospect of developing interventions to promote functional health and for reducing public health expenditures for physical disability in later life.

## Introduction

Although recent trends in the United States show improvements in functional health [1], [2], increased levels of disability remain a major public health problem [3]–[6], especially in later life. Those adults with health risk factors such as being overweight [7], [8], smoking [9], and having alcohol problems [10], as well as those with chronic health conditions [11] are more likely to have poor functional health [12]. A number of socio-demographic variables also are related to functional health [13] including sex [14], [15], race [16]–[18] and socioeconomic status (e.g., educational attainment) [17], [19]. Yet, there is evidence that physical limitations and disability at all ages can be reduced with modifiable lifestyle factors, both by avoiding risk factors [20] and engaging in health-promoting or protective behaviors [21].

Whereas most studies have concentrated on identifying risk factors [22] for negative outcomes such as mortality and morbidity, another approach is to examine the effects of health-promoting factors for maintaining good health, that is focusing on what to do rather than what not to do. This positive approach has been found to be more effective in behavior change for health promotion [23]. There is increasing evidence that specific psychological, social, and physical protective factors are associated with better health in later life [24]–[27]. Among these factors, control beliefs [28]–[31], social support [16], [25], [32], [33], [34], [35], [36], [37] and physical exercise [12], [38], [39], [40], [41], [42], [43] are consistently identified as predictors of functional health. Moreover, a variety of studies have shown that control beliefs [44], social support [45], [46] and physical exercise [47] are modifiable and thus can be subject to interventions to reduce disability and improve functional health.

Control beliefs involve the perception that one can influence what happens in one's life and to what extent one's actions can bring about desired outcomes such as good health. It includes beliefs or expectations about one's abilities and perceptions about external constraints [48]. Stronger beliefs about control over outcomes are associated with better reported health, fewer and less severe symptoms, faster recovery from illness, and higher functional status [49]–[52]. Control beliefs show a pattern of decline in adulthood, making older adults more vulnerable in terms of expectancies about their ability to affect their health [48]. The sense of control is related to functional health, in part, because those who have a higher sense of control are more likely to engage in health-promoting behaviors, such as exercising and eating a healthier diet [53].

Social interactions involve a combination of supportive and stressful experiences. High quality social relationships are those in which support is relatively high and strain relatively low. Social support is associated with health in that those who are socially embedded and experience positive relationships are better off than those who are isolated or involved in strained or stressful relationships [34], [37]. There is also longitudinal evidence for the relationship between positive social exchanges and patterns of physical disability [35], and socially vulnerable elderly are more likely to show disability, frailty, and higher mortality risk [54], [55]. The mechanisms that have been considered include physiological factors such as stress hormones, immune functioning, and inflammatory processes that may be exacerbated for those with low social support or social isolation [33], [54]. Moreover, those who have supportive relationships are more likely to reap the benefits of a more active, engaged and healthy lifestyle [36].

The benefits of physical exercise for health are widely documented [38], [41], [56]. Those who engage in regular exercise are likely to reduce or avoid disability due to the positive effects on, for example, cardiovascular and pulmonary functioning, bone density, and muscle mass [57]. Nevertheless, exercise maintenance is challenging and the long term benefits of exercise interventions have not been conclusively demonstrated.

The benefits of these factors for health have been considered separately in past studies, rather than considered together to examine their unique or combined effects, as we do in the present study. Another key contribution of the current study is to examine the protective contribution of psychological, social, and physical factors to functional health in conjunction with physical risk factors and health status. In a recent study, multiple risk factors were considered individually and in combination in relation to mortality [22], [58], [59]. They found the benefits of a combined measure in that the more of the risk factors present, the greater the mortality risk. However, that study and others focus on the factors that should be avoided, and whether having these factors results in bad outcomes. In contrast, the goal of the present study was to consider the health-promoting role of engaging in psychosocial and behavioral factors and their effects for minimizing declines in functional health. Moreover, we provided a conservative test because we controlled for the effects of health status and traditional physical risk factors to determine if the protective composite would make a difference beyond the factors that many studies have shown to have detrimental effects on health. Thus, the goal of the present study was to examine the combined contribution of the health-promoting variables to long-term changes in health, and to consider their possible protective effect in minimizing aging-related declines in functional health, beyond the role of socio-demographic and physical risk factors.

Psychological, social, and physical protective factors have typically been examined in separate studies. More recently, however, there has been an emphasis on the contribution of multiple factors in relation to health. For example, Kvaavik et al. (2010) [22] showed that the total number of unhealthy risk behaviors in which one person engages, such as smoking, heavy drinking, unhealthy nutrition, and lack of physical activity, was associated with a higher mortality rate. According to Kvaavik et al. (2010, p.711) [22], “to fully understand the public health impact of these behaviors, it is necessary to examine both their individual and combined impact on health outcomes.” The procedure of aggregating different sources of influence stems from the observation that health-related lifestyle factors are not randomly distributed in the general population, but that they tend to occur in combination with each other within individuals [60]. Other examples of this multidimensional approach include computing a cardiovascular risk [61] index or marker of allostatic load [62], which include multiple health indicators and predict disease better than individual indicators. Our goal was to consider the role of multiple factors for maintenance of functional health in middle and older age. Only a modest literature has addressed the relationship between protective psychosocial and behavioral factors and functional limitations [24], [63], [64], [65], [66], and we know of no studies showing their combined effects.

We predicted that, in combination, the protective factors would be associated with functional health over a 10 year period, after controlling for relevant socio-demographic variables, health status, and physical risk factors. We expected that the more of the psychosocial and behavioral factors present at Time 1, the better the maintenance of functional health. We also expected to find evidence for the individual, unique effects of a strong sense of control over life outcomes, high quality of social relationships with friends and family, and frequent vigorous physical exercise on functional health. Previous research has shown that age differences in disability are attenuated by physical exercise [39]. Thus, in the present study we also predicted that the protective factor would serve as a moderator of age differences in functional health. We expected that functional declines would be greater for older adults than for the middle-aged and younger adults, and we examined whether the protective effects would be most beneficial for the older adults. Furthermore, we tested if change in the number of protective factors over time predicted change in functional health while controlling the number of factors at Time 1.

## Materials and Methods

### Ethics Statement

Ethical approval was obtained from the Social and Behavioral Science Institutional Review Board at University of Wisconsin-Madison and by the Committee for Protection of Human Subjects at Brandeis University. All participants gave verbal consent, which included assurance of voluntary participation and confidentiality of data. The ethics committees approved the waiver of written consent. Such passive consent is customary for survey research by telephone and mail questionnaire.

### Sample

Participants were from the Midlife in the United States (MIDUS) survey, conducted in 1995–1996 and 2004–2006, designed to investigate the role of behavioral, social, psychological, biological, and neurological factors in understanding physical and mental health as people age.

A national sample (N = 4,238) from the 48 contiguous states was selected using random-digit dialing (RDD) of telephone numbers with age and sex information about the household composition, with an overall response rate of 70% for the telephone interview [67]. The study also included siblings (N = 949) of the main respondents, randomly selected from the RDD sample, as well as a subpopulation of twins (N = 1,913) obtained after screening a representative national sample of approximately 50,000 households. At Time 1 (N = 7,100) participants ranged in age from 24–75 years (*M* = 46.40, *SD* = 13.00). At Time 2, the longitudinal retention rate, adjusted for mortality, was 75% (N = 4,955), with ages ranging from 32 to 84 years (*M* = 55.45 years, *SD* = 12.44). As typically found, those who participated at the second wave showed positive selection (Table S1) on most variables compared with those who dropped out of the study. For more information on the sample see Radler and Ryff (2010) [68]. When we compared those with complete data who were included in the present analyses (N = 3,626) with those excluded, we found the same pattern of positive selection (Table 1).

**Table 1:** Comparison of the Sample Included in Data Analysis and the Sample Excluded from Data Analysis.

Variable (Time 1)	Included Sample (N = 3,626)	Excluded Sample (N = 3,474)	p value
Age, Mean (SD) in years	47.37 (12.44)	45.37 (13.48)	<.001
Women, %	54.7	48.5	<.001
Education, Mean (SD) in years	14.14 (2.60)	13.34 (2.60)	<.001
Non Hispanic white, %	93.9	86.1	<.001
Health Status	.26 (.53)	.30(.60)	<.001
Waist Circumference, Mean (SD) in inches	35.35 (5.76)	35.49 (5.76)	.372
Do Smoke, %	18.7	27.3	<.001
Do Have Alcohol or Drug Problems, %	2.2	3.1	.026
Control Beliefs, Mean (SD)	5.56 (.99)	5.42 (1.08)	<.001
Quality of Social Support, Mean (SD)	3.18 (.37)	3.13 (.41)	<.001
Physical Exercise, Mean (SD)	4.23 (1.68)	4.01 (1.77)	<.001
Functional Health, Mean (SD)	89.43 (18.80)	83.25 (25.77)	<.001

p values for means are derived from independent samples t-tests.

p values for percentages are derived from χ^2^ tests.

### Measures

#### Functional Health

This measure was based on seven items from the Physical Functioning subscale from the SF-36 Health Survey (α reliability = .90 at Time 1; α reliability = .92 at Time 2) [69]. Three items from the original scale were omitted: one item because it was not measured at Time 1 and the two others because they directly referred to physical exercise, one of our predictors. The seven items, which capture the extent to which the participants' health level limits them in doing different activities (e.g., lifting or carrying groceries, climbing several flights of stairs), were averaged. The same items were used at both times of measurement. The scores ranging from 1 (a lot) to 4 (not at all) were transformed so that the lowest possible score was 0 and the highest possible score 100 [70]. A higher score indicates better health.

#### Protective Factors

We examined three factors from the psychological, social and physical domains.

*Control beliefs* at Time 1 and Time 2 were assessed with a 12-item composite (α reliability Time 1 = .85; Time 2 = .87). This measure of perceived control over outcomes in life was computed by averaging scores on two subscales from the MIDUS sense of control scale [29], namely personal mastery (e.g., I can do just about anything I really set my mind to) and perceived constraints (e.g., What happens in my life is often beyond my control). The scores range from 1 (strongly agree) to 7 (strongly disagree) and were reverse coded for personal mastery. A higher value indicates higher sense of control.

For *quality of social support*, we included items reflecting social support and reverse coded items for social strain for close relationships. This measure (α reliability Time 1 = .87; Time 2 = .88) was the average of the ratings on 12 items assessing socioemotional aspects of social support (e.g., How much do members of your family really care about you) and 12 items assessing the social strain (e.g., How often do members of your family make too many demands on you) that the participants experienced in their relationships with family, friends, and spouse/partner [37], [71]. The scores range from 1 (never) to 4 (often), with a higher value indicating greater quality of social support.

For *physical exercise*, participants reported the frequency of engaging in vigorous physical activities (e.g., running or lifting heavy objects) to work up a sweat, during the summer and winter months at Time 1 [39]. The total physical exercise score throughout the year was the mean of summer and winter ratings, which ranged from 1 (never) to 6 (several times a week or more). At Time 2, six questions assessing the participant's frequency of vigorous physical exercise were used. These questions referred to frequency of physical activities separately for the summer and winter months, in three different settings (i.e., home, work, and leisure), with ratings from 1 (never) to 6 (several times a week). We computed the mean across summer and winter in all three settings and we selected the exercise intensity and setting with the maximum value to represent the highest frequency of physical activity across all domains. At both occasions, higher scores indicate more frequent physical exercise.

To compute the *protective composite* at Time 1 and Time 2, for each factor we assigned the participants a score of 0 (below the median) or 1 (equal to or above the median) for control beliefs (Median Time 1 = 5.75; Time 2 = 5.75), quality of social support (Median Time 1 = 3.21; Time 2 = 3.29), and physical exercise (Median Time 1 = 4.50; Time 2 = 4). The composites were obtained by summing the 3 assigned scores. The total scores ranged from 0 to 3, with higher scores indicating a greater number of factors present at the higher, more desirable level. For comparative purposes, an alternative method was used to compute the composite. In this case we computed z-scores for each of the three factors and summed them to create a continuous linear composite score, as recommended for examining sensitivity in other composites such as allostatic load [72]. The results using this approach (Table S2) were consistent with the median split composite results. For ease of presentation and interpretation, we used the median split method for the analyses presented here.

#### Socio-demographic Variables

We examined *age*, *sex* (−1 =  men, 1 =  women), highest level of *education* in years (ranging from 6 to 20 years), and self-assessed *race* (−1 =  non Hispanic white, 1 =  all others).

#### Health Status

This measure taken at Time 1 assessed how many of the following conditions the participants reported ever having: diabetes, stroke, lupus, HIV/AIDS, multiple sclerosis, epilepsy or other neurological disorders, cancer, or heart trouble (e.g., heart attack). Participants were assigned a score of 1 for any of the chronic conditions. The final score could range from 0 to 7.

#### Physical Risk Factors

The *waist circumference*, a good indicator of obesity that is linked to disability [73], was measured in inches, at the level of the navel, by the participant at Time 1. For the data analysis, this variable was standardized separately for men (*M* = 38.03, *SD* = 4.55) and women (*M* = 33.13, *SD* = 5.71). For *smoking*, participants were asked if they smoke cigarettes regularly now (at Time 1, −1 = no, 1 = yes). For *alcohol or drug problems*, participants reported at Time 1 if they have experienced or have been treated for alcohol or drug problems during the past 12 months (−1 = no, 1 = yes).

### Data Analysis

For the analyses, we included the participants (N = 3,626) who completed all the measures of interest: the protective factors and the covariates at Time 1 and functional health at both occasions. We used hierarchical multiple regression to examine to what extent the individual protective factors and all possible combinations at Time 1 are associated with change in functional health over the 8 to 10 years, and whether the association with age is moderated by the number of protective factors. Although there is much discussion about the best ways to examine change, the residualized change method we used, in which we predicted Time 2 while controlling for the level of health at Time 1, is the most straightforward and widely accepted approach with two occasions of measurement [74]. The regression models also adjusted for the effects of socio-demographic variables, health status, and the physical risk factors. For testing whether change in the number of protective factors over time was related to change in health, we used the difference between the two protective composites (Time 2 level minus Time 1 level) as a predictor, while adjusting for the composite score at Time 1, functional health at Time 1, and covariates. For this analysis, we included the participants with complete longitudinal data on all measures including the three protective factors at both time points (N = 3,578). We also examined a model in which change in the protective factor was converted to a categorical variable (i.e., decrease, stable, increase), controlling for time 1 level, and we found the same results.

As our sample also included siblings of the main respondents and a subpopulation of twins, we also ran models using the cluster option in STATA [75]. This model takes dependencies into account using robust standard errors by clustering at the family level. We found the same results with this more conservative approach. We report the results for both the standard and clustered models in Table 2.

**Table 2:** Hierarchical Multiple Regression with Functional Health at Time 2 as Dependent Variable and with Socio-demographics and Time 1 Variables: Functional Health, Health Status, Physical Risk Factors, and Protective Composite as Predictors.

Predictors	Unstandardized (Standardized) Parameter Estimate	SE (Robust SE†)	p value (p value with Robust SE†)
STEP 1: R^2^ = .411F(6, 3619) = 420.91, p<.001; Clustered F† (6, 2760) = 323.01, p<.001			
Functional Health	.67 (.50)	.018 (.024)	<.001 (<.001)
Age*	−.38 (−.19)	.027 (.028)	<.001 (<.001)
Sex	−1.08 (−.04)	.330 (.327)	.001 (.001)
Education	1.09 (.11)	.128 (.131)	<.001 (<.001)
Race	.23 (.00)	.679 (.760)	.731 (.759)
Health Status	−5.10 (−.11)	.641 (.807)	<.001 (<.001)
**STEP 2: R^2^ change = .024** **F Change (3, 3616) = 51.22, p<.001; Clustered F change** † **(3, 2760) = 37.91, p<.001**			
Functional Health	.61 (.45)	.019 (.025)	<.001 (<.001)
Age*	−.37 (−.18)	.027 (.028)	<.001 (<.001)
Sex	−1.33 (−.05)	.324 (.325)	<.001 (<.001)
Education	.81 (.08)	.129 (.129)	<.001 (<.001)
Race	.24 (.01)	.665 (.748)	.719 (.749)
Health Status	−4.93 (−.10)	.628 (.779)	<.001 (<.001)
Waist Circumference	−3.67 (−.15)	.338 (.388)	<.001 (<.001)
Smoking	−2.61 (−.08)	.426 (.469)	<.001 (<.001)
Alcohol or Drug Problems	−2.51 (−.03)	1.099 (1.442)	.022 (.082)
**STEP 3: R^2^ change = .004** **F Change (1, 3615) = 23.97, p<.001; Clustered F change** † **(1, 2760) = 23.33, p<.001**			
Functional Health	.59 (.44)	.019 (.025)	<.001 (<.001)
Age*	−.37 (−.18)	.027 (.028)	<.001 (<.001)
Sex	−1.19 (−.05)	.324 (.325)	<.001 (<.001)
Education	.76 (.08)	.129 (.129)	<.001 (<.001)
Race	.34 (.01)	.663 (.740)	.609 (.647)
Health Status	−4.83 (−.10)	.627 (.774)	<.001 (<.001)
Waist Circumference	−3.49 (−.14)	.339 (.386)	<.001 (<.001)
Smoking	−2.55 (−.08)	.425 (.467)	<.001 (<.001)
Alcohol or Drug Problems	−2.21 (−.03)	1.097 (1.437)	.044 (.124)
Protective Composite*	1.67 (.06)	.341 (.346)	<.001 (<.001)
**STEP 4: R^2^ change = .001** **F Change (1, 3614) = 4.53, p = .033; Clustered F change** † **(1, 2760) = 3.95, p = .047**			
Functional Health	.59 (.44)	.019 (.026)	<.001 (<.001)
Age*	−.38 (−.18)	.027 (.028)	<.001 (<.001)
Sex	−1.16 (−.05)	.324 (.326)	<.001 (<.001)
Education	.76 (.08)	.128 (.129)	<.001 (<.001)
Race	.34 (.01)	.663 (.740)	.605 (.643)
Health Status	−4.85 (−.10)	.627 (.774)	<.001 (<.001)
Waist Circumference	−3.52 (−.14)	.339 (.387)	<.001 (<.001)
Smoking	−2.58 (−.08)	.425 (.467)	<.001 (<.001)
Alcohol or Drug Problems	−2.28 (−.03)	1.097 (1.431)	.038 (.112)
Protective Composite*	1.69 (.07)	.341 (.347)	<.001 (<.001)
Protective Composite* × Age*	.06 (.03)	.027 (.029)	.033 (.047)

*Age and the protective composite score were centered to the mean.

† Values obtained using cluster option at the family level in STATA.

## Results

The means, standard deviations, and intercorrelations for all variables are presented in Table S3. Over 8 to 10 years, individual differences in functional health [*r*(3576)  = .58, p<.001] and the protective composite [*r*(3576)  = .47, p<.001] were relatively stable, although there was significant average decline for both measures [t(3577)  = 22.72, p<.001; t(3577)  = 2.67, p = .008, respectively]. Correlation analysis revealed that health was negatively associated with age [Time 1: *r*(3576)  = −.21, p<.001; Time 2: *r*(3576)  = −.33, p<.001] and positively related to education level [Time 1: *r*(3576)  = .18, p<.001; Time 2: *r*(3576)  = .23, p<.001]. Men had higher functional health at Time 1 [*r*(3576)  = −.13, p<.001] and Time 2 [*r*(3576)  = −.12, p<.001] than women, and non Hispanic whites had higher functional health at Time 1 [*r*(3576)  = −.07, p<.001] than all others, but race was not significantly related at Time 2 [*r*(3576)  = −.02, p = .211]. Also, in line with previous research, the risk factors (large waist circumference, smoking, and having alcohol or drug problems, respectively), were negatively associated with functional health at Time 1 [*r*(3576)  = −.23, p<.001; *r*(3576)  = −.08, p<.001; *r*(3576)  = −.03, p = .054] and Time 2 [*r*(3576)  = −.26, p<.001; *r*(3576)  = −.10, p<.001; *r*(3576)  = −.05, p = .001]. Moreover, results revealed the expected positive correlations between psychosocial and behavioral factors and health. Higher control beliefs, greater social support, and more frequent physical exercise at Time 1 were associated with better functional health at both occasions of measurement, respectively, [Time 1: *r*(3576)  = .22, p<.001; *r*(3576)  = .11, p<.001; *r*(3576)  = .33, p<.001; Time 2: [*r*(3576)  = .23, p<.001; *r*(3576)  = .09, p<.001; *r*(3576)  = .29, p<.001]. The same relationships with health were obtained with all variables measured at Time 2, between functional health and control beliefs [r(3576)  = .30, p<.001], social support [r(3576)  = .09, p<.001], and physical exercise [r(3576)  = .22, p<.001].

First, we tested a model including all three dichotomous protective factors at Time 1 (low/high levels). While controlling for socio-demographic, health status, and risk factors, all three predictors, high sense of control [B = .03, t(3613)  = 2.40, p = .016], high quality social support [B = .03, t(3613)  = 2.59, p = .010], and frequent physical exercise [B = .03, t(3613)  = 2.50, p = .013] were significantly associated with less decline in functional health. The similar values of the standardized regression coefficients provide a rationale for computing the composite with equal weights for each protective factor.

To test our main hypotheses, we conducted hierarchical multiple regression to examine predictors of residualized change in functional health, with and without using the robust standard errors (Table 2). The dependent variable was Time 2 functional health, and at the first step we controlled for Time 1 functional health, socio-demographic predictors, and health status; all were statistically significant and accounted for 41% of the variance [F(6, 3619)  = 420.91, p<.001]. Consistent with past work, older age was associated with greater decline in functional health. In the second step, introducing the Time 1 physical risk factors led to a significant increase in the percentage of variance [R^2^ change = .024, F Change (3, 3616)  = 51.22, p<.001].

**Figure 1:**
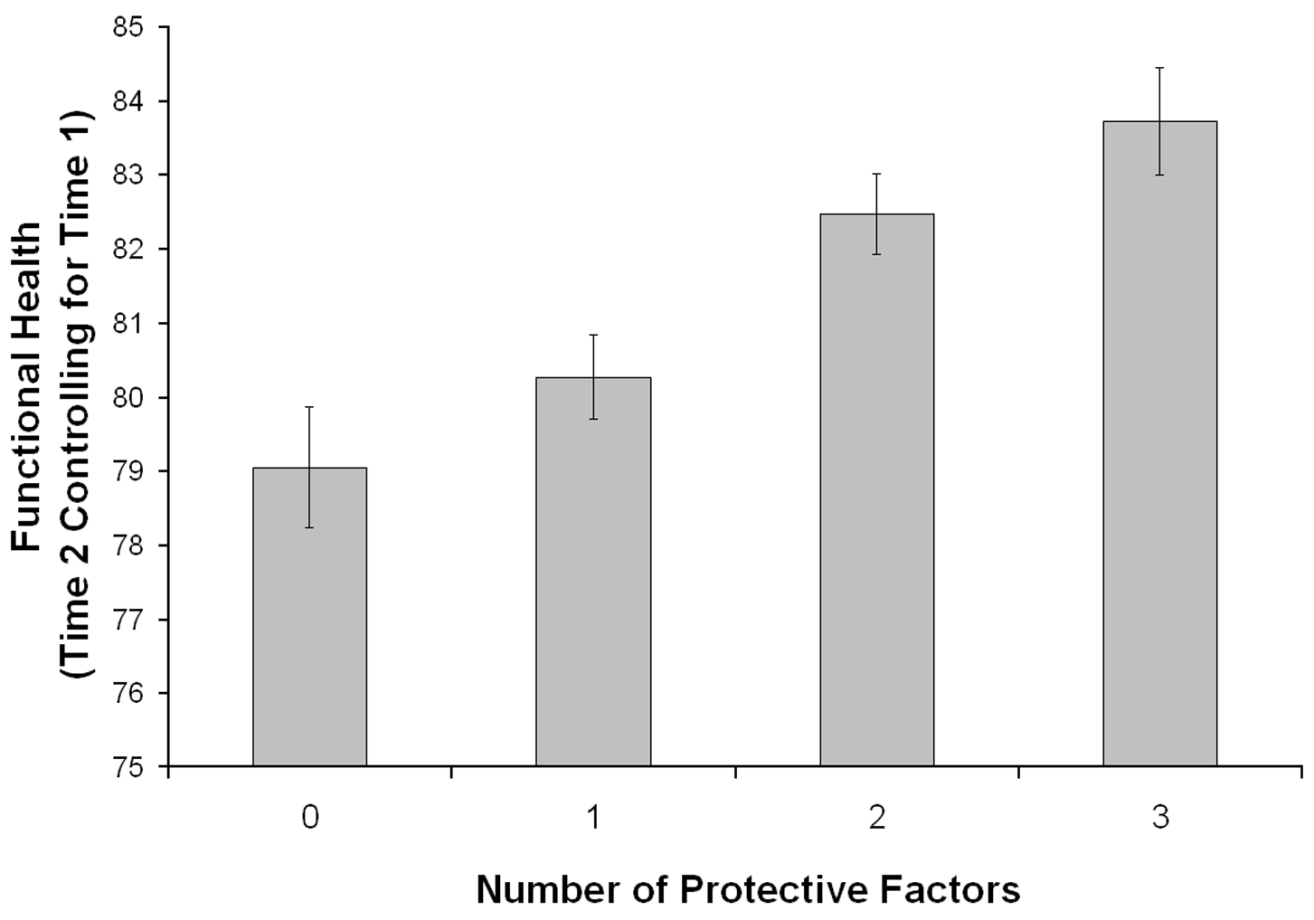
The Estimated Means for Residual Change in Functional Health as a Function of the Number of Time 1 Protective Factors, Adjusted for Socio-demographics, Health Status, Physical Risk Factors, and Functional Health at Time 1, Derived from an ANCOVA Model [F(3, 3613) = 8.17, p<.001]. Errors bars are standard errors of the mean.

At the third step, when the 3 protective factors were entered as an aggregate score, the composite had a significant effect [B = .06, t(3615)  = 4.90, p<.001] on change in functional health, over and above the role of socio-demographic, health status, and risk factors, and added significant additional variance [R^2^ change = .004, F Change (1, 3615)  = 23.97, p<.001]. The more behavioral factors one has, the greater the maintenance of health over 8 to 10 years (Figure 1). This was also illustrated by the percentages of explained variance obtained in a complementary model (Table S4), including each of the protective factors individually (steps 3.1a, 3.1b, 3.1c), in combinations of two (steps 3.2a, 3.2b, 3.2c), as well as the above mentioned three-factor composite (step 3.3). Moreover, all the individual protective factors explained a significant percent of variance, over and above the role of the socio-demographics, health status, and risk factors. The same was true for all possible pairs of protective factors, which, as indicated by the percentages of explained variance (R^2^ change  = .003), had an equal contribution to change in functional health. Further analyses also showed that, in all cases, adding a third factor resulted in a significant increase in R^2^ over and above each of the two factor combinations.

**Figure 2:**
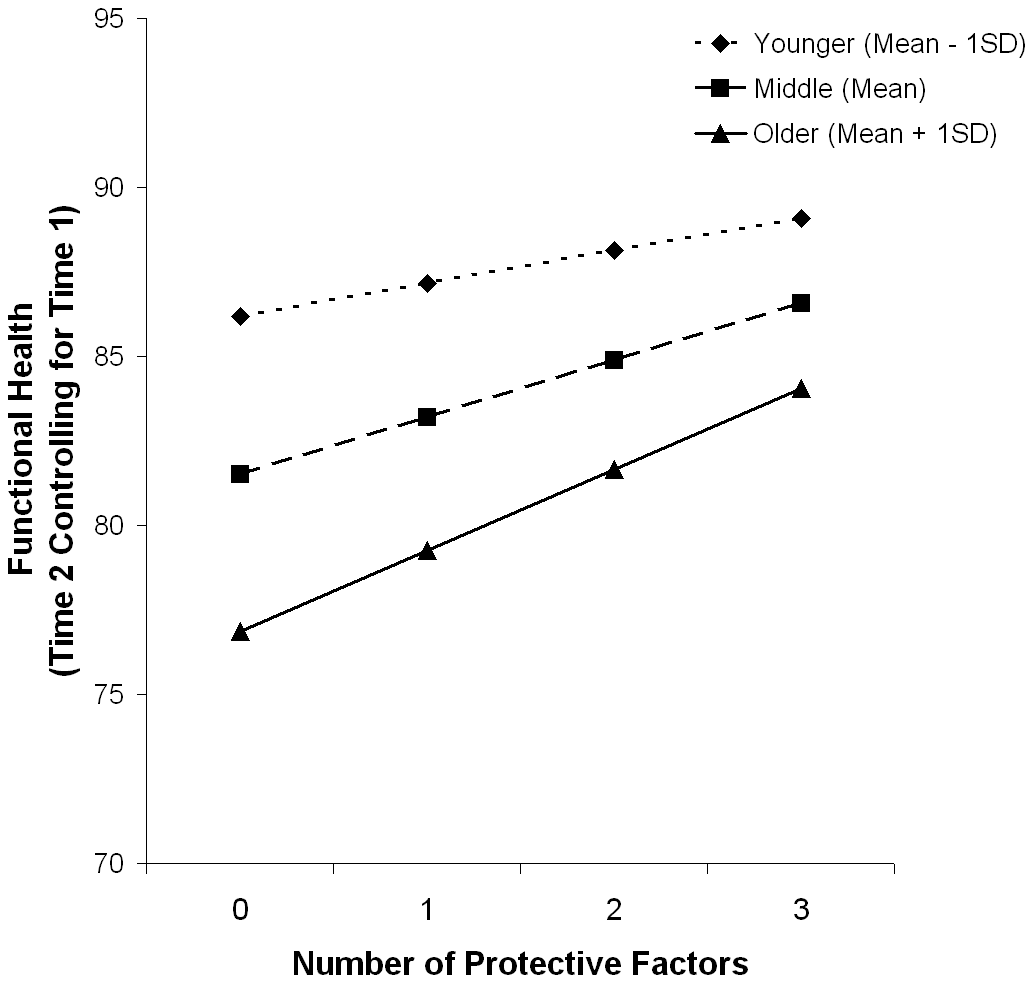
The Predicted Values for Residual Change in Functional Health by Age at Time 2 and Number of Protective Factors at Time 1, Adjusted for Socio-demographics, Health Status, Physical Risk Factors, and Functional Health at Time 1; Age Time 2: *M* = 56.30, *SD* = 12.39.

Also using multiple regression, we examined whether the effect of the 3-factor composite on change in functional health varied by age. A significant age by composite interaction showed that the difference in functional health between younger and older adults was significantly reduced with an increased number of protective factors [Figure 2, B = .03, t(3614)  = 2.13, p = .033].

We also examined whether change in the number of protective factors over time was related to maintenance or change in functional health. While controlling for the Time 1 composite, functional health, socio-demographic variables, and physical risk factors, the greater the increase in the number of factors the better the health at Time 2 [B = .09, t(3566)  = 6.22, p<.001]. In this model, the number of protective factors at Time 1 remained significantly associated with functional health [B = .12, t(3566)  = 7.35, p<.001].

## Discussion

The results provide support for the role of modifiable psychological, social, and physical protective factors, in addition to minimizing physical risk factors, as a means for reducing physical disability. On average, functional health declined significantly over the 8 to 10 years and, as expected, the declines were greatest in later life. Yet, those who engaged in multiple factors were protected in that they showed better maintenance of functional health. Whereas previous research has typically examined one of these factors at a time {e.g., [54], [56]}, we found that each of the factors had a unique independent contribution, with or without adjusting for the other factors. Moreover, the results show evidence for the additive value of these factors, and the more of them the better in terms of health outcomes. Although the psychosocial and behavioral factors were beneficial for health at all ages, a key promising finding is that age differences in functional health were significantly attenuated and health declines were reduced as a function of the number of protective factors present at adaptive levels 8 to 10 years earlier. This is consistent with previous findings on the potential of protective factors such as physical exercise to reduce age differences in functional health [39]. The protective factors were found to have a differentially beneficial effect at the oldest ages that is, among those who are most at risk and vulnerable to functional declines. Among older adults who had all 3 protective factors at adaptive levels, their functional health was more comparable with the level of those in the younger and middle-aged groups. The relationship between protective factors at Time 1 and functional health at Time 2 is significant when controlling for Time 1 functional health, illustrating that the effects are related to long term maintenance or change in health, independent of initial level of health. Moreover, engaging in more protective factors over time was positively related to functional health, independent of the number of behaviors people adopted earlier in life.

Some limitations of the study should be noted, especially the restricted measurement of some variables (e.g., smoking, alcohol and drug problems, physical exercise). We used self-report assessments for all variables, and the relationships could be affected by a common measurement bias. Nevertheless, the psychosocial variables are typically measured using self report, given the focus on perceptions and expectancies. Moreover, the functional health measure has been well validated in relation to objective indicators of disability in previous work [76]. In the case of both health and exercise, more objective measures (e.g., pedometers for physical exercise) may be examined in future studies. Also, the generalizability of findings must be considered in light of the positive selection of the longitudinal sample, although the large probability base for the sample is an asset. Although the present study design with two waves of measurement enabled us to examine changes in health and protective factors, including three or more occasions will allow for consideration of the multidirectionality and consistency of these patterns using more sophisticated analytic models [77].

The study shows the importance of considering long-term predictors of functional health, with a goal of reducing disability, improving quality of life, and reducing health care costs. Some groups may be more susceptible to health problems and less likely to engage in the protective factors, such as those who are older or those with lower socioeconomic status (SES) [24]. Acknowledging these vulnerabilities could lead to targeted interventions to reduce age and SES differences in health. The lifestyle factors in midlife (e.g., the 50's) showed a protective effect for health in later life (e.g., the 60's) [78] and the more of them the better. The promising findings raise possibilities of examining the joint effects of other psychosocial and behavioral factors such as stress reactivity and regulation [79], spirituality and religious activity [80], nutrition and diet [81], and to investigate whether there would be continued value added. Moreover, future work is needed to explore the mechanisms {e.g., stress hormones [82], inflammation [83], allostatic load [84]} that may be involved in linking the protective factors to health.

The findings suggest that multifaceted psychosocial and behavioral interventions that target multiple components such as a sense of control, good quality social relationships with family and friends, and physical exercise in early adulthood and midlife could have a dramatic protective effect in reducing disability and maintaining functional health and independence into later life [85], over and above the contribution of reducing physical risk factors [20], [21], [24], [26]. One implication of the findings is that multidimensional interventions may be more effective than ones focused on single dimensions for improving health quality of life. An example is a clinical trial successful in reducing disability among sedentary older adults [41], [86], by focusing on multiple factors: improving the sense of control over exercise, increasing physical strength with resistance training, and providing social support from an exercise trainer. This type of multimodal intervention is likely to result in higher compliance and better maintenance than studies that rely only on changing physical activity without the psychological mindset and social support needed to promote long-term behavioral changes. Given the increasing expenditures on health care for those over age 65 with physical limitations during the last decade [6], the results are encouraging for the prospect of reducing public health expenditures for physical disability in later life.

## Supporting Information

Table S1 Comparison of the Longitudinal Participants and the Dropouts.
(0.02 MB PDF)

Table S2 Hierarchical Multiple Regression with Functional Health at Time 2 as Dependent Variable and with Socio-demographics and Time 1 Variables: Functional Health, Health Status, Physical Risk Factors, and Protective Composite (computed with continuous z-scores) as Predictors.
(0.02 MB PDF)

Table S3 Means, Standard Deviations, and Intercorrelations for all Variables.
(0.03 MB PDF)

Table S4 Hierarchical Multiple Regression with Functional Health at Time 2 as Dependent Variable and with Socio-demographics and Time 1 Variables: Functional Health, Health Status, Physical Risk Factors, and all Combinations of Protective Factors as Predictors.
(0.02 MB PDF)
