# Supplementary material for: Promoting Functional Health in Midlife and Old Age: Long-Term Protective Effects of Control Beliefs, Social Support, and Physical Exercise
Source: PLoS One. 2010 Oct 11;5(10):e13297. doi: 10.1371/journal.pone.0013297 (PMC2952603; doi:10.1371/journal.pone.0013297)
Supplement: Table S1 — Comparison of the Longitudinal Participants and the Dropouts. (0.02 MB PDF) [file pone.0013297.s001.pdf]

Table S1. Comparison of the Longitudinal Participants and the Dropouts

Variable (Time 1)	Longitudinal (N = 4,955)	Dropouts (N = 2,145)	p value
Age, Mean (SD) in years	46.48 (12.50)	46.21 (14.10)	.457
Women, %	53.3	47.8	< .001
Education, Mean (SD) in years	14.02 (2.60)	13.13 (2.60)	< .001
Non Hispanic white, %	93.0	84.0	< .001
Health Status	.26 (.53)	.33 (.64)	< .001
Waist Circumference, Mean (SD) in inches	35.33 (5.75)	35.63 (5.79)	.079
Do Smoke, %	19.6	30.6	< .001
Do Have Alcohol or Drug Problems, %	2.2	3.6	.002
Control Beliefs, Mean (SD)	5.54 (1.00)	5.38 (1.09)	< .001
Quality of Social Support, Mean (SD)	3.18 (.37)	3.13 (.43)	< .001
Physical Exercise, Mean (SD)	4.20 (1.69)	3.96 (1.81)	< .001
Functional Health, Mean (SD)	88.73 (19.98)	81.38 (26.93)	< .001

p values for means are derived from independent samples t-tests

p values for percentages are derived from χ^2 tests
