# Supplementary material for: Promoting Functional Health in Midlife and Old Age: Long-Term Protective Effects of Control Beliefs, Social Support, and Physical Exercise
Source: PLoS One. 2010 Oct 11;5(10):e13297. doi: 10.1371/journal.pone.0013297 (PMC2952603; doi:10.1371/journal.pone.0013297)
Supplement: Table S2 — Hierarchical Multiple Regression with Functional Health at Time 2 as Dependent Variable and with Socio-demographics and Time 1 Variables: Functional Health, Health Status, Physical Risk Factors, and Protective Composite (computed with continuous z-scores) as Predictors. (0.02 MB PDF) [file pone.0013297.s002.pdf]

**Table S2. Hierarchical Multiple Regression with Functional Health at Time 2 as Dependent Variable and with Socio-demographics and Time 1 Variables: Functional Health, Health Status, Physical Risk Factors, and Protective Composite (computed with continuous z-scores) as Predictors**

| Predictors | Unstandardized (Standardized) Parameter Estimate | SE (Robust SE†) | p value (p value with Robust SE†) |
|---|---|---|---|
| **STEP 1 : $R^2$ = .411** | | | |
| **F(6, 3619) = 420.91, p < .001; Clustered F† (6, 2760) = 323.01, p < .001** | | | |
| Functional Health | .67 (.50) | .018 (.024) | < .001 (< .001) |
| Age* | -.38 (-.19) | .027 (.028) | < .001 (< .001) |
| Sex | -1.08 (-.04) | .330 (.327) | .001 (.001) |
| Education | 1.09 (.11) | .128 (.131) | < .001 (< .001) |
| Race | .23 (.00) | .679 (.760) | .731 (.759) |
| Health Status | -5.10 (-.11) | .641 (.807) | < .001 (< .001) |
| **STEP 2 : $R^2$ change = .024** | | | |
| **F Change (3, 3616) = 51.22, p < .001; Clustered F change† (3, 2760) = 37.91, p < .001** | | | |
| Functional Health | .61 (.45) | .019 (.025) | < .001 (< .001) |
| Age* | -.37 (-.18) | .027 (.028) | < .001 (< .001) |
| Sex | -1.33 (-.05) | .324 (.325) | < .001 (< .001) |
| Education | .81 (.08) | .129 (.129) | < .001 (< .001) |
| Race | .24 (01) | .665 (.748) | .719 (.749) |
| Health Status | -4.93 (-.10) | .628 (.779) | < .001 (< .001) |
| Waist Circumference | -3.67 (-.15) | .338 (.388) | < .001 (< .001) |
| Smoking | -2.61 (-.08) | .426 (.469) | < .001 (< .001) |
| Alcohol or Drug Problems | -2.51 (-.03) | 1.099 (1.442) | .022 (.082) |
| **STEP 3 : $R^2$ change = .006,** | | | |
| **F Change (1, 3615) = 38.55, p < .001; Clustered F change† (1, 2760) = 31.47, p < .001** | | | |
| Functional Health | .58 (.43) | .019 (.026) | < .001 (< .001) |
| Age* | -.38 (-.18) | .027 (.028) | < .001 (< .001) |
| Sex | -1.12 (-.04) | .324 (.326) | .001 (.001) |
| Education | .74 (.08) | .128 (.129) | < .001 (< .001) |
| Race | .42 (.01) | .662 (.735) | .529 (.571) |
| Health Status | -4.79 (-.10) | .626 (.772) | < .001 (< .001) |
| Waist Circumference | -3.44 (-.14) | .338 (.387) | < .001 (< .001) |
| Smoking | -2.55 (-.08) | .424 (.466) | < .001 (< .001) |
| Alcohol or Drug Problems | -2.04 (-.02) | 1.096 (1.431) | .063 (.154) |
| Protective Composite* | 1.03 (.08) | .165 (.183) | < .001 (< .001) |

**Table S2 (continued)**

| STEP 4 : $R^2$ change = .001<br>F Change (1, 3614) = 5.00, p = .025; Clustered F change[†] (1, 2760) = 3.88, p =.049 | | | |
|---|---|---|---|
| Functional Health | .57 (.43) | .019 (.026) | < .001 (< .001) |
| Age* | -.38 (-.19) | .027 (.028) | < .001 (< .001) |
| Sex | -1.09 (-.04) | .324 (.327) | .001 (.001) |
| Education | .74 (.08) | .128 (.129) | < .001 (< .001) |
| Race | .38 (.01) | .662 (.733) | .564 (.602) |
| Health Status | -4.78 (-.10) | .625 (.772) | < .001 (< .001) |
| Waist Circumference | -3.48 (-.14) | .339 (.388) | < .001 (< .001) |
| Smoking | -2.58 (-.08) | .424 (.466) | < .001 (< .001) |
| Alcohol or Drug Problems | -2.14 (-.03) | 1.096 (1.425) | .051 (.133) |
| Protective Composite* | 1.02 (.08) | .165 (.182) | < .001 (< .001) |
| Protective Composite* x Age* | .03 (.03) | .013 (.015) | .025 (.049) |

*Age and the protective composite score were centered to the mean
[†] Values obtained using cluster option at the family level in STATA
