# Supplementary material for: Promoting Functional Health in Midlife and Old Age: Long-Term Protective Effects of Control Beliefs, Social Support, and Physical Exercise
Source: PLoS One. 2010 Oct 11;5(10):e13297. doi: 10.1371/journal.pone.0013297 (PMC2952603; doi:10.1371/journal.pone.0013297)
Supplement: Table S3 — Means, Standard Deviations, and Intercorrelations for all Variables. (0.03 MB PDF) [file pone.0013297.s003.pdf]

Table S3. Means, Standard Deviations, and Intercorrelations for all Variables

	M	*SD*	1	2	3	4	5	6	7
1. Age Time 1 (years)	47.27	12.39							
2. Sex (-1 = men, 1 = women)	-	-	-.02						
3. Education Time 1 (years)	14.16	2.59	-.09*	-.13*					
4. Race (-1 = white, 1 = other)	-	-	-.05*	.05*	-.01				
5. Health Status Time 1	.25	.53	.24*	.01	-.03	-.03			
6. Waist Circumference Time 1 (inches)	35.36	5.74	.19*	-.42*	-.07*	.00	.10*		
7. Smoking Time 1 (-1 = no, 1 = yes)	-	-	-.12*	.01	-.21*	.00	-.01	-.06*	
8. Alcohol or Drug Problems Time 1 (-1 = no, 1 = yes)	-	-	-.02	-.05*	.00	.01	.05*	.02	.14*
9. Control Beliefs Time 1	5.56	.98	-.06*	-.07*	.16*	-.02	-.09*	-.08*	-.02
10. Quality of Social Support Time 1	3.18	.37	.15*	.03	.02	-.06*	-.03	-.08*	-.06*
11. Physical Exercise Time 1	4.23	1.67	-.21*	-.25*	.16*	-.06*	-.10*	-.09*	-.07*
12. Protective Composite Time 1	1.57	.97	-.05*	-.12*	.15*	-.05*	-.09*	-.11*	-.07*
13. Control Beliefs Time 2	5.55	.99	-.05*	-.08*	.15*	-.03	-.10*	-.06*	-.03
14. Quality of Social Support Time 2	3.24	.37	.20*	.01	.03	-.08*	-.02	-.05*	-.06*
15. Physical Exercise Time 2	3.74	1.85	-.32*	-.12*	.16*	-.06*	-.09*	-.07*	-.02
16. Protective Composite Time 2	1.53	.95	-.07*	-.08*	.14*	-.06*	-.09*	-.08*	-.04[†]
17. Functional Health Time 1	89.58	18.59	-.21*	-.13*	.18*	-.07*	-.24*	-.23*	-.08*
18. Functional Health Time 2	81.67	25.19	-.33*	-.12*	.23*	-.02	-.28*	-.26*	-.10*

Table S3 (continued)

	8	9	10	11	12	13	14	15	16	17
1. Age Time 1 (years)										
2. Sex (-1 = men, 1 = women)										
3. Education Time 1 (years)										
4. Race (-1 = white, 1 = other)										
5. Health Status Time 1										
6. Waist Circumference Time 1 (inches)										
7. Smoking Time 1 (-1 = no, 1 = yes)										
8. Alcohol or Drug Problems Time 1 (-1 = no, 1 = yes)										
9. Control Beliefs Time 1	-.05*									
10. Quality of Social Support Time 1	-.10*	.38*								
11. Physical Exercise Time 1	.00	.18*	.05*							
12. Protective Composite Time 1	-.06*	.64*	.59*	.53*						
13. Control Beliefs Time 2	-.05*	.60*	.30*	.18*	.44*					
14. Quality of Social Support Time 2	-.11*	.32*	.61*	.05*	.39*	.41*				
15. Physical Exercise Time 2	.01	.11*	-.06*	.36*	.17*	.12*	-.08*			
16. Protective Composite Time 2	-.06*	.44*	.35*	.26*	.47*	.65*	.54*	.47*		
17. Functional Health Time 1	-.03	.22*	.11*	.33*	.26*	.24*	.09*	.17*	.20*	
18. Functional Health Time 2	-.05*	.23*	.09*	.29*	.24*	.30*	.09*	.22*	.24*	.58*

Table S3 (continued)

*Correlation is significant at the 0.01 level (2-tailed)
[†]Correlation is significant at the 0.05 level (2-tailed)
N = 3,578; *M* = Mean; *SD* = Standard Deviation
Age ranges from 24 to 75 years. Education level ranges from 6 to 20 years. For the health status, the number of chronic conditions ranges from 0 to 6. Waist circumference in inches ranges from 20 to 66 for women and 26.5 to 61 for men. Control beliefs range from 1.08 to 7 at both times. Quality of social support ranges from 1.44 to 4 at Time 1 and from 1.58 to 4 at Time 2. Physical exercise ranges from 1 to 6 at both times. Both protective composites range from 0 to 3. Both measures of functional health range from 0 to 100.
