# Supplementary material for: Promoting Functional Health in Midlife and Old Age: Long-Term Protective Effects of Control Beliefs, Social Support, and Physical Exercise
Source: PLoS One. 2010 Oct 11;5(10):e13297. doi: 10.1371/journal.pone.0013297 (PMC2952603; doi:10.1371/journal.pone.0013297)
Supplement: Table S4 — Hierarchical Multiple Regression with Functional Health at Time 2 as Dependent Variable and with Socio-demographics and Time 1 Variables: Functional Health, Health Status, Physical Risk Factors, and all Combinations of Protective Factors as Predictors. (0.02 MB PDF) [file pone.0013297.s004.pdf]

Table S4. Hierarchical Multiple Regression with Functional Health at Time 2 as Dependent Variable and with Socio-demographics and Time 1 Variables: Functional Health, Health Status, Physical Risk Factors, and all Combinations of Protective Factors as Predictors

	Predictors	Unstandardized (Standardized) Parameter Estimate	SE (Robust SE*)	p value (p value with Robust SE*)
**Step 1**	**R^2 = .411**<br>**F(6, 3619) = 420.91, p < .001; Clustered F* (6, 2760) = 323.01, p < .001**			
	Functional Health	.67 (.50)	.018 (.024)	< .001 (< .001)
	Age	-.38 (-.19)	.027 (.028)	< .001 (< .001)
	Sex	-1.08 (-.04)	.330 (.327)	.001 (.001)
	Education	1.09 (.11)	.128 (.131)	< .001 (< .001)
	Race	.23 (.00)	.679 (.760)	.731 (.759)
	Health Status	-5.10 (-.11)	.641 (.807)	< .001 (< .001)
**Step 2**	**R^2 change = .024**<br>**F change (3, 3616) = 51.22, p < .001; Clustered F change* (3, 2760) =37.91, p < .001**			
	Functional Health	.61 (.45)	.019 (.025)	< .001 (< .001)
	Age	-.37 (-.18)	.027 (.028)	< .001 (< .001)
	Sex	-1.33 (-.05)	.324 (.325)	< .001 (< .001)
	Education	.81 (.08)	.129 (.129)	< .001 (< .001)
	Race	.24 (.01)	.665 (.748)	.719 (.749)
	Health Status	-4.93 (-.10)	.628 (.779)	< .001 (< .001)
	Waist Circumference	-3.67 (-.15)	.338 (.388)	< .001 (< .001)
	Smoking	-2.61 (-.08)	.426 (.469)	< .001 (< .001)
	Alcohol or Drug Problems	-2.51 (-.03)	1.099 (1.442)	.022 (.082)

		Unstandardized (Standardized) Parameter Estimate	SE (Robust SE*)	R^2 change	F change (1, 3615) (Clustered F change* (1, 2760))	p value (p value with Robust SE*)
**Step 3.1a**	Control Beliefs	2.14 (.04)	.646 (.660)	.002	10.94 (10.48)	.001 (.001)
**Step 3.1b**	Social Support	2.15 (.04)	.645 (.639)	.002	11.06 (11.27)	.001 (.001)
**Step 3.1c**	Physical Exercise	1.86 (.04)	.680 (.699)	.001	7.47 (7.07)	.006 (.008)

Table S4 (continued)

		Unstandardized (Standardized) Parameter Estimate	SE (Robust SE*)	R^2 change	F change (1, 3615) (Clustered F change* (1, 2760))	p value (p value with Robust SE*)
**Step 3.2a**	(Control Beliefs & Social Support)	1.72 (.05)	.409 (.416)	.003	17.72 (17.12)	< .001 (< .001)
**Step 3.2b**	(Social Support & Physical Exercise)	1.97 (.06)	.463 (.461)	.003	18.10 (18.20)	< .001 (< .001)
**Step 3.2c**	(Control Beliefs & Physical Exercise)	1.87 (.06)	.452 (.462)	.003	17.13 (16.40)	< .001 (< .001)
**Step 3.3**	Protective Composite (Control Beliefs & Social Support & Physical Exercise)	1.67 (.06)	.341 (.346)	.004	23.97 (23.33)	< .001 (< .001)

* Values obtained using cluster option at the family level in STATA
